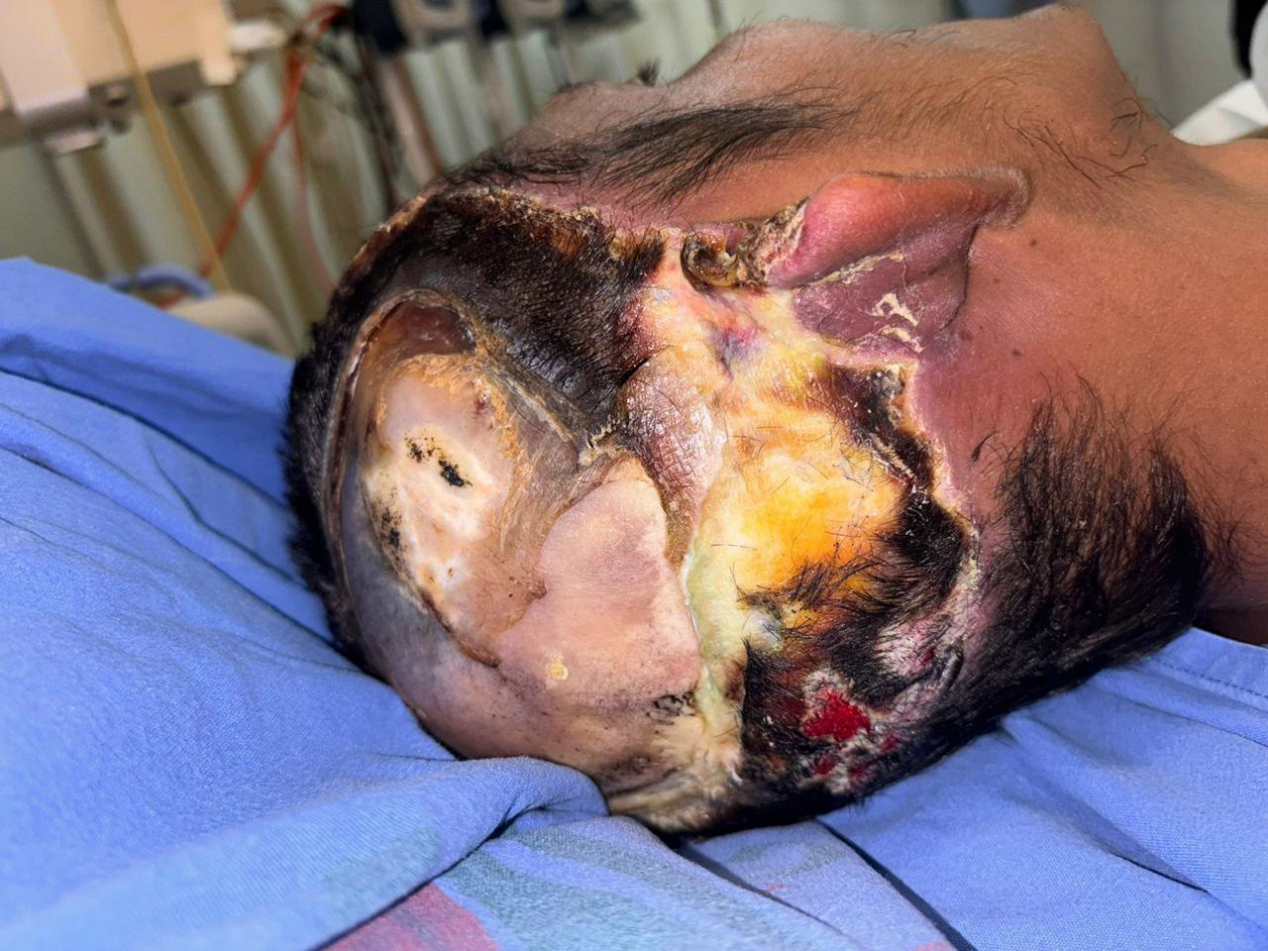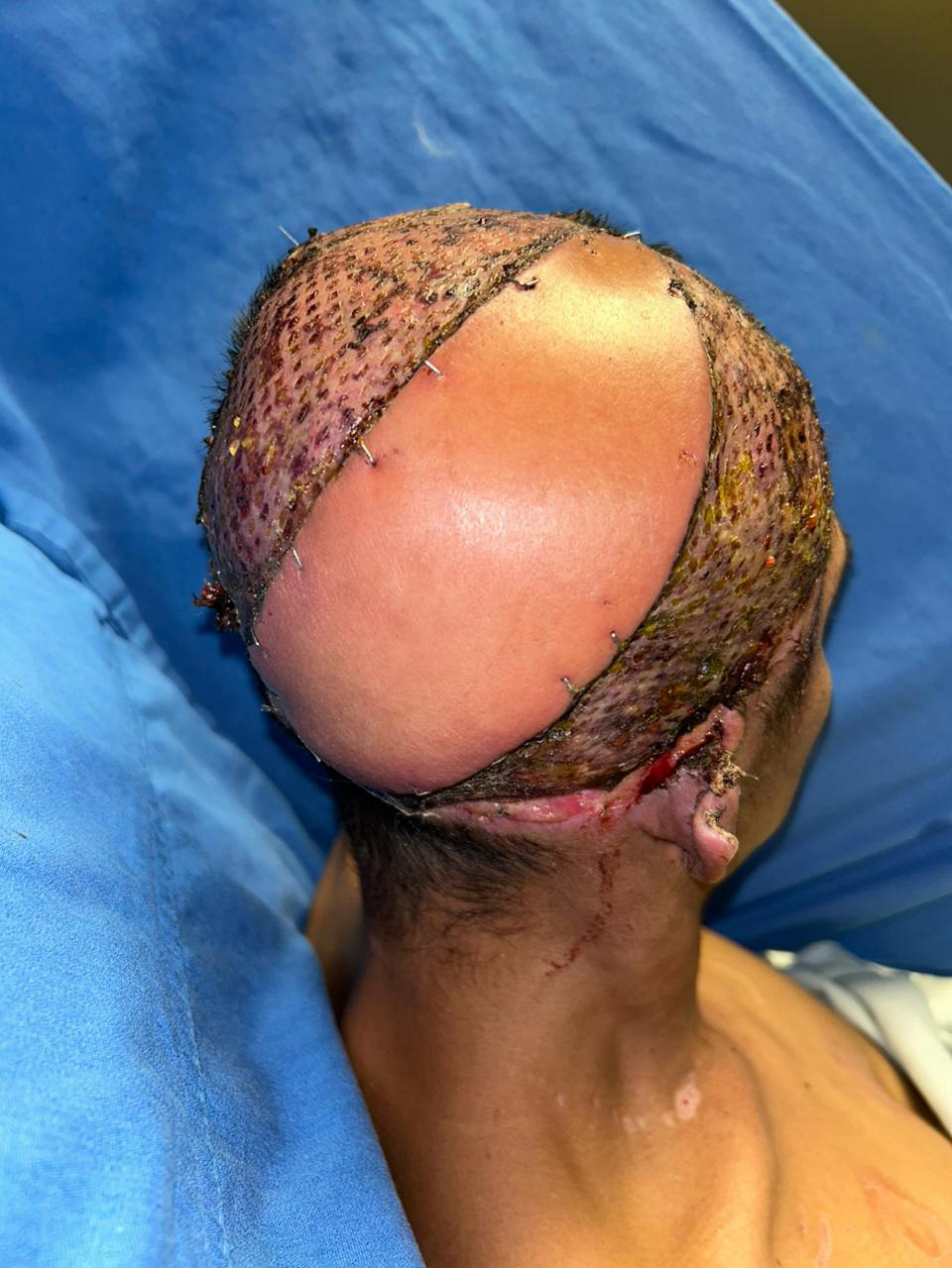# 991 Free Lattissimus Dorsi Flap for Large Scalp Defects Reconstruction: Case Report and Surgical Technique

**DOI:** 10.1093/jbcr/iraf019.522

**Published:** 2025-04-01

**Authors:** Lucia Cano Pérez, Luis Tamez Pedroza

**Affiliations:** Christus Muguerza Alta Especialidad; Instituto Nacional de Rehabilitación

## Abstract

**Introduction:**

The free latissimus dorsi flap for scalp or skull reconstruction, is a versatile solution for a wide range of reconstructive challenges. The latissimus dorsi muscle is a large, triangular back muscle with fibers form de 7th to the 12th thoracic vertebrae, its blood supply is delivered from the subscapular artery branch called the thoracodorsal artery. In this case the function of the remaining latissimus dorsi muscle is preserved. The posterior axillary fold is also preserved through the remaining cephalic portion of the muscle, resulting in an improved aesthetic outcome postoperatively. Preserving the posterior part of the latissimus dorsi and its innervation also improves symmetry and the contour of the back, avoiding the asymmetric back.

**Methods:**

We present a 30-year-old male patient with 5% electrical burn of the skull with temporal, parietal and right occipital involvement, with third degree burn and bone exposure in these areas. He suffered contact of the temporal area of the head with a high voltage cable, losing consciousness and without apparent cranioencephalic trauma; however, with loss of consciousness for 5 minutes. He was refered to the emergency room of our Hospital “Instituto Nacional de Rehabilitación ‘‘ in Mexico City, Mexico. During his hospitalization he was admitted to ICU and went to the OR for debridement of necrotic tissue. On physical examination he had bone exposure of the temporal, parietal and occipital bone in the right hemicranium, with presence of soft tissue necrosis with a diameter of 20X5 cm and involvement of the aesthetic unit of the ear. The patient had a negative pressure system prior evaluated for reconstruction. Surgical reconstruction was performed using a latissimus dorsi free flap micro anastomosed to the superficial temporal vessels.The patient was seen again 7 days later, he was well with no evidence of flap necrosis. He was followed up for an additional six months with good evolution.

**Results:**

We present a successful case of a patient who was reconstructed with a Free Lattissimus dorsi flap.

**Conclusions:**

With its numerous advantages, the function-preserving split latissimus dorsi muscle flap provides an excellent option as a free flap for skull reconstruction.

**Applicability of Research to Practice:**

The goals of reconstructive surgery for the burn patient are to restore function and aesthetic appearance. The goals of scalp reconstruction are to provide full coverage of the calvarium to prevent desiccation, sequestration, infection, and cover the defect with hair-bearing skin. Vascular damage caused to the vessels by electricity includes blood vessel occlusion and endothelial cell death which continues increasing, from 30 min to days after burn, causing a cessation of blood flow, which makes preferable to look for a receiving vessel that have better condition than the nearest one.

**Funding for the Study:**

N/A